# Supplementary figures and images for: Empathy and its associations with age and sociodemographic characteristics in a large UK population sample
Source: PLoS One. 2021 Sep 20;16(9):e0257557. doi: 10.1371/journal.pone.0257557 (PMC8452078; doi:10.1371/journal.pone.0257557)

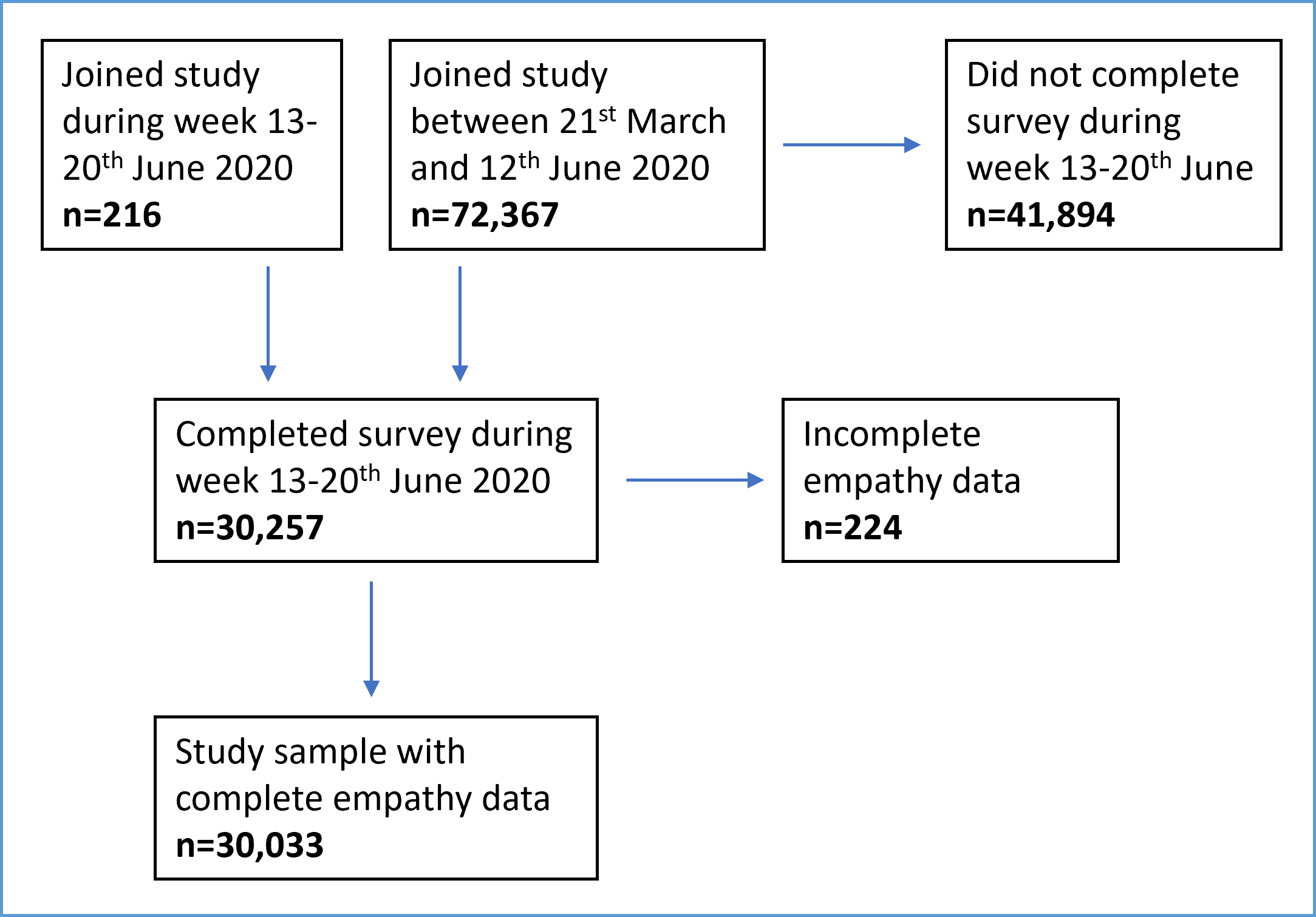

Supplement: S1 Fig — Empathy questions were included in the study during week 13-20th June 2020. (TIF) [file pone.0257557.s001.tif]
