# Supplementary material for: Empathy and its associations with age and sociodemographic characteristics in a large UK population sample
Source: PLoS One. 2021 Sep 20;16(9):e0257557. doi: 10.1371/journal.pone.0257557 (PMC8452078; doi:10.1371/journal.pone.0257557)
Supplement: S1 Table — sd = standard deviation; acategorised into tertiles based on distribution in this sample. (DOCX) [file pone.0257557.s002.docx]

## S1 Table. Mean unweighted scores on empathic concern and perspective taking scales according to sociodemographic characteristics (n=25,169)

|  |  | Empathic concern | | | Perspective taking | |
| --- | --- | --- | --- | --- | --- | --- |
|  |  | Mean | sd | Mean | | sd |
| Age (years) | 18-25 | 4.01 | 0.67 | 3.71 | | 0.68 |
|  | 25-34 | 4.00 | 0.65 | 3.70 | | 0.65 |
|  | 35-44 | 4.00 | 0.66 | 3.72 | | 0.66 |
|  | 45-54 | 4.02 | 0.68 | 3.71 | | 0.69 |
|  | 55-64 | 3.97 | 0.67 | 3.62 | | 0.71 |
|  | 65-74 | 3.91 | 0.65 | 3.62 | | 0.69 |
|  | ≥75 | 3.87 | 0.63 | 3.55 | | 0.69 |
| Gender | Male | 3.70 | 0.66 | 3.48 | | 0.69 |
|  | Female | 4.06 | 0.64 | 3.74 | | 0.68 |
|  | Other / prefer not to say | 3.95 | 0.64 | 3.49 | | 0.64 |
| Ethnicity | White | 3.97 | 0.66 | 3.68 | | 0.69 |
|  | Other | 4.02 | 0.67 | 3.66 | | 0.69 |
| Educational level | Lower secondary | 3.90 | 0.69 | 3.54 | | 0.73 |
|  | Higher secondary | 3.94 | 0.68 | 3.64 | | 0.71 |
|  | Graduate | 3.99 | 0.65 | 3.71 | | 0.67 |
| Living status | Alone | 3.93 | 0.67 | 3.64 | | 0.70 |
|  | With others | 3.98 | 0.66 | 3.68 | | 0.69 |
| Marital status | Single | 3.91 | 0.68 | 3.62 | | 0.69 |
|  | Divorced/widowed | 4.00 | 0.67 | 3.71 | | 0.71 |
|  | Non cohabiting partner | 3.99 | 0.67 | 3.70 | | 0.66 |
|  | Married/cohabiting | 3.97 | 0.66 | 3.68 | | 0.68 |
| Employment | Not working | 3.94 | 0.66 | 3.63 | | 0.71 |
|  | Working | 3.99 | 0.66 | 3.71 | | 0.67 |
| Household income | < £30,000 | 3.97 | 0.67 | 3.66 | | 0.71 |
|  | ≥ £30,000 | 3.97 | 0.65 | 3.69 | | 0.67 |
| ‘Keyworker’ status | None of these | 3.95 | 0.66 | 3.66 | | 0.69 |
|  | Health/social-care | 4.11 | 0.64 | 3.80 | | 0.67 |
|  | Teacher/childcare | 4.13 | 0.65 | 3.79 | | 0.67 |
|  | Other ‘keyworker’ | 3.91 | 0.67 | 3.65 | | 0.68 |
| Carer status | Not carer | 3.95 | 0.66 | 3.66 | | 0.69 |
|  | carer | 4.04 | 0.65 | 3.73 | | 0.69 |
| Face-to-face social contact | < 1 time per week | 3.90 | 0.69 | 3.62 | | 0.70 |
|  | 1-2 times per week | 3.97 | 0.66 | 3.68 | | 0.68 |
|  | 3+ times per week | 4.02 | 0.64 | 3.71 | | 0.69 |
| Long-term condition | No | 3.96 | 0.66 | 3.69 | | 0.67 |
|  | Yes | 3.97 | 0.66 | 3.65 | | 0.71 |
| Neuroticism ^a^ | Low | 3.90 | 0.67 | 3.74 | | 0.68 |
|  | Medium | 3.95 | 0.65 | 3.67 | | 0.67 |
|  | High | 4.07 | 0.65 | 3.60 | | 0.70 |
| Extroversion ^a^ | Low | 3.86 | 0.69 | 3.61 | | 0.69 |
|  | Medium | 3.98 | 0.64 | 3.68 | | 0.67 |
|  | High | 4.10 | 0.63 | 3.75 | | 0.69 |
| Openness to experience ^a^ | Low | 3.82 | 0.67 | 3.56 | | 0.69 |
|  | Medium | 3.98 | 0.63 | 3.69 | | 0.66 |
|  | High | 4.14 | 0.64 | 3.81 | | 0.69 |
| Agreeableness ^a^ | Low | 3.69 | 0.66 | 3.36 | | 0.67 |
|  | Medium | 4.00 | 0.60 | 3.72 | | 0.61 |
|  | High | 4.27 | 0.59 | 4.00 | | 0.63 |
| Conscientiousness ^a^ | Low | 3.87 | 0.65 | 3.56 | | 0.67 |
|  | Medium | 3.99 | 0.64 | 3.71 | | 0.66 |
|  | High | 4.12 | 0.69 | 3.75 | | 0.72 |

**Notes:** sd = standard deviation; ^a^categorised into tertiles based on distribution in this sample
