# Supplementary material for: Empathy and its associations with age and sociodemographic characteristics in a large UK population sample
Source: PLoS One. 2021 Sep 20;16(9):e0257557. doi: 10.1371/journal.pone.0257557 (PMC8452078; doi:10.1371/journal.pone.0257557)
Supplement: S2 Table — Linear regression models. Multivariable models are mutually adjusted for included variables. Coefficients indicate estimated difference in Interpersonal reactivity index empathic concern score according to respondent characteristic. (DOCX) [file pone.0257557.s003.docx]

## S2 Table. Association of participant characteristics with empathic concern – unweighted univariable and multivariable associations (n=25,169)

|  |  | Unweighted univariable  Complete cases | | Unweighted multivariable  Complete cases | |
| --- | --- | --- | --- | --- | --- |
|  |  | Coefficient | P value | Coefficient | P value |
| Age (years) | 18-25 | Reference | <0.001 | Reference | 0.003 |
|  | 25-34 | -0.02 (-0.10, 0.05) |  | 0.01 (-0.06, 0.07) |  |
|  | 35-44 | -0.03 (-0.10, 0.04) |  | 0.04 (-0.03, 0.10) |  |
|  | 45-54 | -0.01 (-0.08, 0.05) |  | 0.07 (0.00, 0.13) |  |
|  | 55-64 | -0.06 (-0.13, 0.01) |  | 0.04 (-0.03, 0.10) |  |
|  | 65-74 | -0.12 (-0.19, -0.05) |  | 0.02 (-0.04, 0.09) |  |
|  | ≥75 | -0.15 (-0.22, -0.07) |  | 0.03 (-0.04, 0.10) |  |
| Gender | Male | Reference | <0.001 | Reference | <0.001 |
|  | Female | 0.36 (0.34, 0.38) |  | 0.22 (0.20, 0.24) |  |
| Ethnicity | White | Reference | 0.02 | Reference | 0.001 |
|  | Other | 0.05 (0.01, 0.10) |  | 0.06 (0.02, 0.10) |  |
| Educational level | Lower secondary | Reference | <0.001 | Reference | 0.001 |
|  | Higher secondary | 0.04 (0.01, 0.07) |  | 0.00 (-0.02, 0.03) |  |
|  | Graduate | 0.08 (0.06, 0.11) |  | 0.03 (0.01, 0.06) |  |
| Living | Alone (ref) | Reference | <0.001 | Reference | 0.02 |
|  | With others | 0.06 (0.04, 0.08) |  | 0.03 (0.01, 0.06) |  |
| Marital status | Single (ref) | Reference | <0.001 | Reference | 0.10 |
|  | Divorced/widowed | 0.09 (0.06, 0.12) |  | 0.03 (0.00, 0.06) |  |
|  | Non cohabiting partner | 0.08 (0.04, 0.12) |  | 0.01 (-0.02, 0.05) |  |
|  | Married/cohabiting | 0.06 (0.04, 0.09) |  | 0.03 (0.00, 0.06) |  |
| Employment | Not working (ref) | Reference | <0.001 | Reference | 0.21 |
|  | Working | 0.05 (0.03, 0.07) |  | -0.01 (-0.03, 0.01) |  |
| Household income | < £30,000 (ref) | Reference | 0.51 | Reference | 0.98 |
|  | ≥ £30,000 | 0.01 (-0.01, -0.02) |  | -0.00 (-0.02, 0.02) |  |
| ‘Keyworker’ status | None of these (ref) | Reference | <0.001 | Reference | <0.001 |
|  | Health/social-care | 0.16 (0.13, 0.19) |  | 0.10 (0.08, 0.13) |  |
|  | Teacher/childcare | 0.18 (0.14, 0.23) |  | 0.06 (0.02, 0.10) |  |
|  | Other ‘keyworker’ | -0.05 (-0.08, -0.02) |  | -0.01 (-0.04, 0.01) |  |
| Carer status | Not carer (ref) | Reference | <0.001 | Reference | 0.05 |
|  | carer | 0.09 (0.07, 0.12) |  | 0.02 (0.00, 0.04) |  |
| Face-to-face social contact | < 1 time per week (ref) | Reference | <0.001 | Reference | <0.001 |
|  | 1-2 times per week | 0.08 (0.06, 0.10) |  | 0.03 (0.01, 0.05) |  |
|  | 3+ times per week | 0.12 (0.09, 0.14) |  | 0.03 (0.01, 0.05) |  |
| Long-term condition | No (ref) | Reference | 0.01 | Reference | <0.001 |
|  | Yes | 0.02 (0.01, 0.04) |  | 0.03 (0.02, 0.05) |  |
| Personality mean score  (per one standard deviation higher) | Neuroticism | 0.07 (0.07, 0.08) | <0.001 | 0.10 (0.10, 0.11) | <0.001 |
|  | Extroversion | 0.11 (0.10, 0.12) | <0.001 | 0.06 (0.05, 0.07) | <0.001 |
|  | Openness to experience | 0.14 (0.13, 0.15) | <0.001 | 0.11 (0.11, 0.12) | <0.001 |
|  | Agreeableness | 0.26 (0.25, 0.27) | <0.001 | 0.24 (0.23, 0.24) | <0.001 |
|  | Conscientiousness | 0.09 (0.09, 0.10) | <0.001 | 0.00 (-0.01, 0.01) | 0.50 |

**Notes:** Linear regression models. Multivariable models are mutually adjusted for included variables. Coefficients indicate estimated difference in Interpersonal reactivity index empathic concern score according to respondent characteristic.
