# Supplementary material for: Empathy and its associations with age and sociodemographic characteristics in a large UK population sample
Source: PLoS One. 2021 Sep 20;16(9):e0257557. doi: 10.1371/journal.pone.0257557 (PMC8452078; doi:10.1371/journal.pone.0257557)
Supplement: S3 Table — Linear regression models, mutually adjusted for included variables, and weighted to the UK proportions of gender, age, ethnicity, education and country of living obtained from the Office for National Statistics. Coefficients indicate estimated difference in Interpersonal reactivity index empathic concern or perspective taking score according to respondent characteristic. (DOCX) [file pone.0257557.s004.docx]

## S3 Table: Multivariable weighted associations of participant characteristics with empathic concern or perspective taking with missing data imputed using multiple imputation (n=30,033)

|  |  | Empathic concern | | Perspective taking | |
| --- | --- | --- | --- | --- | --- |
|  |  | Coefficient | P value | Coefficient | P value |
| Age (years) | 18-25 | Reference | 0.13 | Reference | <0.001 |
|  | 25-34 | 0.00 (-0.08, 0.08) |  | -0.02 (-0.11, 0.07) |  |
|  | 35-44 | 0.01 (-0.08, 0.09) |  | -0.01 (-0.10, 0.08) |  |
|  | 45-54 | 0.04 (-0.04, 0.12) |  | -0.06 (-0.15, -0.03) |  |
|  | 55-64 | 0.05 (-0.03, 0.13) |  | -0.09 (-0.18, -0.00) |  |
|  | 65-74 | 0.06 (-0.02, 0.14) |  | -0.09 (-0.19, -0.00) |  |
|  | ≥75 | 0.04 (-0.05, 0.13) |  | -0.20 (-0.30, -0.10) |  |
| Gender | Male | Reference | <0.001 | Reference | <0.001 |
|  | Female | 0.22 (0.20, 0.25) |  | 0.18 (0.16, 0.21) |  |
|  | Other/prefer not to say | 0.26 (0.11, 0.41) |  | 0.12 (-0.01, 0.25) |  |
| Ethnicity | White | Reference | 0.004 | Reference | 0.81 |
|  | Other | 0.08 (0.03, 0.14) |  | 0.01 (-0.05, 0.07) |  |
| Educational level | Lower secondary | Reference | 0.005 | Reference | <0.001 |
|  | Higher secondary | 0.01 (-0.02, 0.04) |  | 0.06 (0.03, 0.09) |  |
|  | Graduate | 0.04 (0.01, 0.07) |  | 0.09 (0.06, 0.12) |  |
| Living | Alone (ref) | Reference | 0.84 | Reference | 0.63 |
|  | With others | 0.00 (-0.04, 0.04) |  | -0.01 (-0.05, 0.03) |  |
| Marital status | Single (ref) | Reference | 0.08 | Reference | 0.30 |
|  | Divorced/widowed | 0.04 (-0.00, 0.08) |  | 0.04 (-0.00, 0.08) |  |
|  | Non cohabiting partner | 0.04 (-0.01, 0.10) |  | 0.04 (-0.02, 0.10) |  |
|  | Married/cohabiting | 0.05 (0.01, 0.09) |  | 0.02 (-0.01, 0.08) |  |
| Employment | Not working (ref) | Reference | 0.92 | Reference | 0.67 |
|  | Working | -0.00 (-0.03, 0.03) |  | 0.01 (-0.02, 0.04) |  |
| Household income | < £30,000 (ref) | Reference | 0.78 | Reference | 0.35 |
|  | ≥ £30,000 | -0.00 (-0.03, 0.02) |  | 0.02 (-0.01, 0.04) |  |
| ‘Keyworker’ status | None of these (ref) | Reference | <0.001 | Reference | 0.02 |
|  | Health/social-care | 0.11 (0.06, 0.15) |  | 0.05 (0.02, 0.10) |  |
|  | Teacher/childcare | 0.06 (0.00, 0.11) |  | -0.03 (-0.10, 0.03) |  |
|  | Other ‘keyworker’ | -0.01 (-0.05, 0.04) |  | 0.02 (-0.03, 0.06) |  |
| Carer status | Not carer (ref) | Reference | 0.82 | Reference | 0.17 |
|  | carer | 0.00 (-0.03, 0.03) |  | 0.02 (-0.00, 0.05) |  |
| Face-to-face social contact | < 1 time per week (ref) | Reference | 0.02 | Reference | 0.52 |
|  | 1-2 times per week | 0.03 (-0.00, 0.05) |  | 0.02 (-0.01, 0.05) |  |
|  | 3+ times per week | 0.04 (0.01, 0.07) |  | 0.01 (-0.02, 0.04) |  |
| Long-term condition | No (ref) | Reference | 0.26 | Reference | 0.79 |
|  | Yes | 0.01 (-0.01, 0.04) |  | -0.00 (-0.03, 0.02) |  |
| Personality mean score  (per one standard deviation higher) | Neuroticism | 0.11 (0.10, 0.12) | <0.001 | -0.07 (-0.08, -0.05) | <0.001 |
|  | Extroversion | 0.06 (0.05, 0.07) | <0.001 | -0.01 (-0.02, 0.01) | 0.39 |
|  | Openness to experience | 0.11 (0.10, 0.12) | <0.001 | 0.09 (0.07, 0.10) | <0.001 |
|  | Agreeableness | 0.24 (0.23, 0.26) | <0.001 | 0.26 (0.24, 0.27) | <0.001 |
|  | Conscientiousness | 0.00 (-0.01, 0.01) | 0.96 | 0.01 (-0.00, 0.02) | 0.16 |

**Notes:** Linear regression models, mutually adjusted for included variables, and weighted **to the UK proportions of gender, age, ethnicity, education and country of living obtained from the Office for National Statistics.** Coefficients indicate estimated difference in Interpersonal reactivity index empathic concern or perspective taking score according to respondent characteristic.
